# Socioeconomic inequality in intermittent preventive treatment using Sulphadoxine pyrimethamine among pregnant women in Nigeria

**DOI:** 10.1186/s12889-020-09967-w

**Published:** 2020-12-04

**Authors:** Fatima Mahmud Muhammad, Reza Majdzadeh, Saharnaz Nedjat, Haniye Sadat Sajadi, Mahboubeh Parsaeian

**Affiliations:** 1 Department of Epidemiology & Biostatistics, School of Public Health, Tehran University of Medical Sciences, Tehran, Iran; 2 Department of Epidemiology & Biostatistics, Knowledge Utilization Research Center and Community-Based Participatory-Research-Center, School of Public Health, Tehran University of Medical Sciences, Tehran, Iran; 3 Knowledge Utilization Research center, University Research and Development Center, Tehran University of Medical Sciences, Tehran, Iran

**Keywords:** Malaria, Intermittent preventive treatment, Socioeconomic inequality, Decomposition analysis, Nigeria

## Abstract

**Background:**

Intermittent preventive treatment using Sulphadoxine pyrimethamine (IPTp-SP) for malaria prevention is recommended for all pregnant women in malaria endemic areas. However, there is limited evidence on the level of socioeconomic inequality in IPTp-SP use among pregnant women in Nigeria. Thus, this study aimed to determine the level of socioeconomic inequality in IPTp-SP use among pregnant women in Nigeria and to decompose it into its contributing factors.

**Methods:**

A secondary data analysis of Nigerian demographic and health survey of 2018 was conducted. A sample of 21,621 pregnant women aged between 15 and 49 years and had live birth in the previous 2 years before the survey were included in this analysis. The study participants were recruited based on a stratified two-stage cluster sampling method. Socioeconomic inequality was decomposed into its contributing factors by concentration index.

**Result:**

Totally 63.6% of pregnant women took at least one dose of IPTp-SP prophylaxis. Among IPTp-SP users, 35.1% took one dose, 38.6% took two doses and 26.2% took three doses and more**.** Based on both concentration index of 0.180 (*p*-value = < 0.001, 95% CI: 0.176 to 0.183) and Erreyger’s normalization concentration index 0.280 (p-value = < 0.001, 95% CI: 0.251 to 0.309), the IPTp-SP utilization was pro-rich. The largest contributors to the inequality in IPTp-SP uptake were wealth index (47.81%) and educational status (28.66%).

**Conclusion:**

Our findings showed that IPTp-SP use was pro-rich in Nigeria. Wealth index and educational status were the factors that significantly contributed to the inequality. The disparities could be reduced through free IPTp service expansion by targeting pregnant women from low socioeconomic status.

**Supplementary Information:**

The online version contains supplementary material available at 10.1186/s12889-020-09967-w.

## Background

Malaria is one of the most public health problems, especially among children and pregnant women in low and middle income countries [1]. An estimated 11 million pregnant women across 38 countries in the sub-Saharan Africa region were infected with malaria in 2018 [2]. This makes malaria infection during pregnancy a significant public health problem (i.e., 29% of all pregnancies) [2], with substantial risks like maternal anemia, placental accumulation of parasite, low birth weight (LBW) and intrauterine growth retardation (IUGR), congenital infection and infant mortality (IM) [3, 4].

Nigeria is one of the countries bearing the highest burden of malaria. The prevalence of malaria in pregnant women in the country ranges from 19.7 to 72.0% [5, 6]. Further, it is a major cause of morbidity and mortality in the country [7].

The World Health Organization (WHO) recommends Intermittent Preventive Treatment in pregnancy (IPTp) in moderate to high malaria transmission areas in Africa [4]. However, considerable proportion of pregnant women do not use IPTp in Nigeria. For example, according to the Malaria indicator survey of 2018 revealed that 64% of women took IPTp [5].

Previous study conducted in Nigeria revealed a connection between malaria and poverty [6]. For example, women living in poorest household are less likely to report increased use of IPT compared to women from the richest household [8, 9]. As a result, Inequity in health service is unacceptable and unfair [10] and should be removed through free service expansion to the underserved population. Inequality is frequently assessed based on socioeconomic status that is measured in asset-based wealth quintiles, residence, sex, age and ethnicity [11].

While recent studies have reported inequalities related to malaria prevention especially in the use and ownership of mosquito nets, [12, 13], little exist explaining the inequalities in relation to the IPTp use among pregnant women. In this study, we assessed the factors contributing to the inequality in IPTp-SP use among pregnant women in Nigeria, and decomposed the socioeconomic inequality into its contributing factors. We hope findings of the study will contribute to reversing the burden of malaria in Nigeria, and beyond especially in the WHO African Region which carries a disproportionately greater share of the global malaria burden.

## Methods

### Study design and participants

The data for this study is the 2018 Nigeria Demographic and Health Survey (DHS) which is the fifth and the most recent survey implemented in the country by the DHS. Data collection was done from 14 August to 29 December 2018.The sampling frame used for the cross-sectional survey was generated from the results of the 2006 Population and Housing Census of Nigeria, the most recent census of Nigeria. The sample was subsequently selected using a stratified two-stage cluster design. Stratification was achieved by separating each of the 36 states and the Federal Capital Territory into urban and rural areas. In the first stage, 1400 enumeration areas were selected randomly with probability proportional to the enumeration area size. The primary sampling unit (PSU), referred to as a cluster, is defined on the basis of enumeration areas from the 2006 census frame. In each cluster, 30 households were randomly selected and all the women aged 15–49 who are either permanent residents or visitors present in the households were eligible to be interviewed. A questionnaire was used to interview all eligible women. Totally 21,621 women responded to question on IPTp use during pregnancy 2 years preceding the survey. Thus these women were used for the analysis of this study. More information on the survey methodology is available in the NDHS 2018 final report [14].

### Variable definition

The outcome variable of this study was use of IPTp at least one dose among pregnant women. Independent variables included in the analysis were age, educational status, marital status, place of residence, wealth index, region, number of antenatal care visits and parity (see Supplementary Table 1). The wealth index is a composite measure of a household’s cumulative living standard constructed using principal component analysis (PCA) as a proxy for socioeconomic status. Households were given scores based on the number and kinds of consumer goods they own, ranging from a television to a bicycle or car, and housing characteristics such as source of drinking water, toilet facilities, and flooring materials. The wealth index ranks each person in the population by their score and then divides the ranking into five equal parts, from quintile one (lowest-poorest) to quintile five (highest-wealthiest), each having approximately 20% of the population. Details could be accessed at DHS guide [15].

### Statistical analysis

The main measures of inequality were concentration curve and concentration index [16]. The concentration curve shows the cumulative percentage of the health outcome (IPTp use) on the y-axis against cumulative percentage of the population in the x-axis, ranked by the wealth index from the poorest to the richest. The curve will appear linear if all women irrespective of their wealth status have exactly the same value of the IPTp use. When the curve lies below the diagonal line it is pro-rich and above it indicates the pro poor inequality. The concentration index is defined as twice the area between the concentration curve and the line of equality (the 45-degree line).

Since the outcome variable was binary we used the Erreyger’s normalized concentration index as preferred over the conventional concentration index [17]. Refer below:

$$ E(h)=4=\frac{\mu }{b_h-{a}_h}\ \mathrm{C}\left(\mathrm{h}\right) $$

Where *b*_*h*_ and *a*_*h*_ are the maximum and minimum of the health variable.

### Decomposition analysis

Decomposition analysis method was proposed by Wagstaff et al. [16] to break down the socioeconomic inequality into its determinants. It also estimates how determinants proportionally contribute to inequality (e.g. the gap between poor and rich) in a health variable. We applied a generalized linear model for binomial distribution with identity link function for linking IPTp intake (y) to the set of k determinants (X_k_) [18] because it considers the structure of IPTp. By this method, the estimates do not vary with the choice of the reference group (yes or no). These render the model appropriate for our analysis. Decomposition analysis was used to break down the use of IPTp-SP. Due to the non-proportional allocation of the sample to the different states and possible difference in response rate, all analyses where weighted and adjusted for the design effect. Statistical analysis was conducted using STATA version 14. Significance level was set at *p* ≤ 0.05.

## Result

**Fig. 1:**
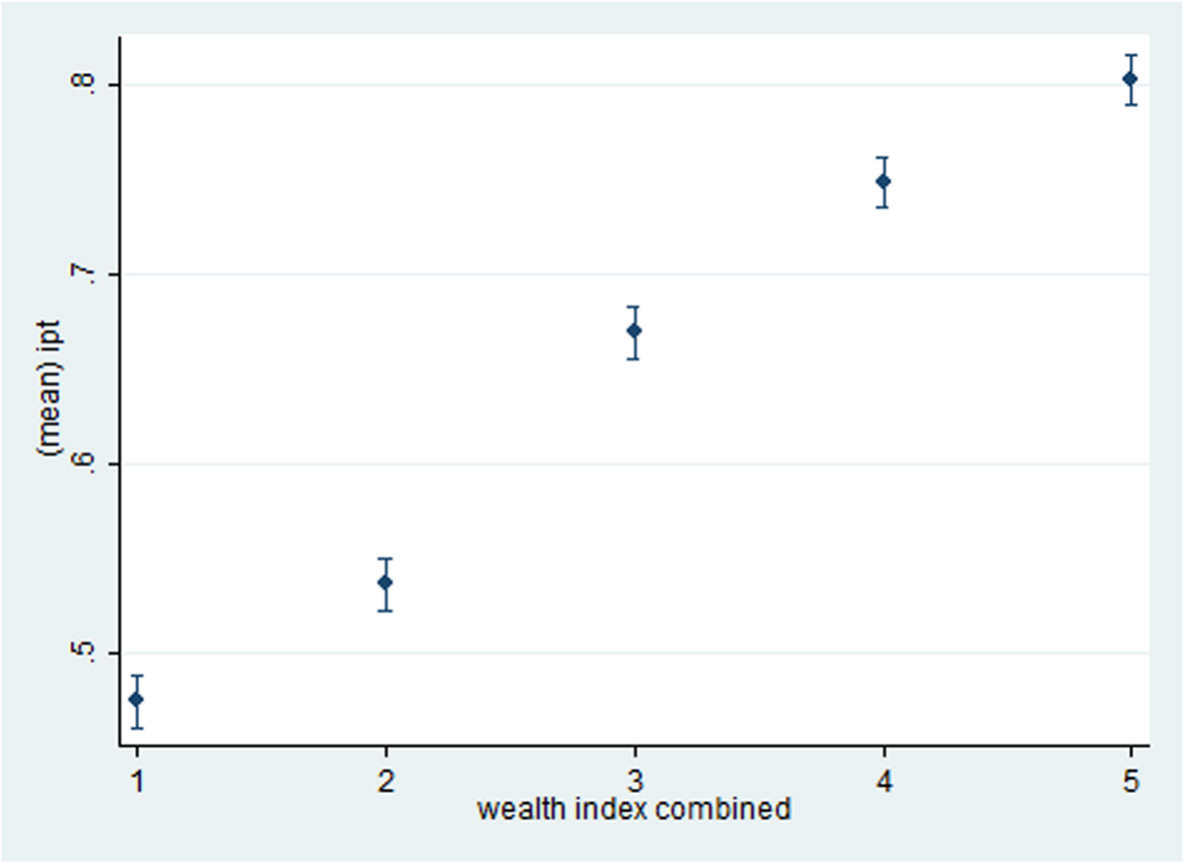
Mean intake of Intermittent preventive treatment using Sulphadoxine Pyrimethamine (IPTp-SP) among pregnant women in Nigeria, by household wealth index 2018. Note: IPT refers to Intermittent Preventive Treatment

The socio-demographic characteristics of respondents are given in Table 1. The mean age of the respondents was 29 years (SD = 9.70). The majority of the respondents (64.7%) were rural dwellers and 9498 (43.9%) with no education. Eighty-nine percent of the respondents received IPTp from antenatal care. The overall use of IPTp-SP among the women was 63.6%. Among IPTp users, 35.1% took one dose, 38.6% took two doses and 26.2 took three doses and more**.** The adjusted associations between IPTp use and its determinants are shown in Table 2. Women with higher educational status [Adjusted OR (95% CI): 1.35 (1.14 to 1.60) *p*-value = < 0.001], being from the richest quintile [Adjusted OR (95% CI): 2.32(1.99 to 2.72) *p*-value = < 0.001], attending 4 or more antenatal visits [Adjusted OR (95% CI): 14.66 (13.42 to 16.02) increased the probability of IPTp use. Age group, place of residency, parity and marital status showed no significant association. The Mean intake of Intermittent preventive treatment using Sulphadoxine Pyrimethamine (IPTp-SP) is presented in Fig. 1.

**Table 1:** Socio-demographic characteristics of women aged 15–49 years who had a live birth 2 years preceding the survey, by intake of one dose of 1PTp

Variables		N (%)	IPT^§^ n (%)	
			Yes	No
Age group	15–24	5362 (24.79)	3222 (60.09)	2140 (39.91)
	25–34	10,197 (47.16)	6607 (64.79)	3590 (35.21)
	35–44	5357 (24.77)	3453 (64.46)	1904 (35.54)
	≥45	705 (3.26)	423 (60.00)	282 (40.00)
Educational status	No education	9498 (43.92)	4862 (51.18)	4636 (48.81)
	Primary	3373 (15.60)	2232 (66.17)	5162 (33.83)
	Secondary	6973 (32.25)	5162 (74.03)	1811 (25.97)
	Higher	1777 (8.21)	1449 (81.54)	328 (18.46)
Marital Status	Never Married	600 (2.77)	378 (63.00)	222 (37.00)
	Married	20,259 (93.70)	12,821 (63.29)	7438 (36.71)
	Separated	762 (3.52)	506 (66.40)	256 (33.60)
Place of residence	Urban	7634 (35.30)	5559 (72.82)	2075 (27.18)
	Rural	13,987 (64.69)	8146 (58.24)	5841 (41.76)
Wealth index	Poorest	5007 (23.15)	2368 (47.29)	2639 (52.71)
	Poorer	4865 (22.50)	2686 (55.21)	2179 (44.79)
	Middle	4549 (21.03)	3072 (67.53)	1477 (32.47)
	Richer	3986 (18.43)	2990 (75.01)	996 (24.99)
	Richest	3214 (14.86)	2589 (80.55)	625 (19.45)
Region	North central	3844 (17.77)	2245 (58.40)	1599 (41.60)
	North east	4502 (20.82)	2827 (62.79)	1675 (37.21)
	North west	6297 (29.12)	3678 (58.41)	2619 (41.59)
	South east	2340 (10.82)	1827 (78.08)	513 (21.92)
	South south	2108 (9.74)	1481 (70.26)	627 (29.74)
	South west	2530 (11.70)	1647 (65.010)	883 (34.90)
ANC^a^ visits	No visits	5630 (26.03)	1158 (20.57)	4472 (79.43)
	< 4 times	3778 (17.47)	2745 (72.66)	1033 (27.34)
	≥4 times	12,213 (56.48)	9802 (80.26)	2411 (19.74)
Parity	1 child	3678 (17.01)	2410 (65.52)	1268 (34.48)
	2 children	3750 (17.34)	2430 (64.80)	1320 (35.20)
	≥ 3 children	14,193 (65.64)	8865 (62.46)	5328 (37.53)

^a^ *ANC* Antenatal Care, ^§^ *IPT* Intermittent preventive treatment

**Table 2:** Bivariate and Multivariable logistic regression of socio-demographic characteristics and risk factors among women aged 15–49 years who had a live birth 2 years preceding the survey, by the intake of IPT, NDHS 2018

Variable		Crude OR(95% CI)	*P* value	Adjusted OR(95% CI)	*P* value
Age group	15–24	1		1	
	25–34	1.22 (1.14 to 1.31)	< 0.001	1.03 (0.93 to 1.13)	0.57
	35–44	1.20 (1.11 to 1.30)	< 0.001	1.09 (0.97 to 1.23)	0.14
	≥45	0.99 (0.85 to 1.17)	0.960	1.07 (0.87 to 1.32)	0.53
Educational status	No education	1		1	
	Primary	1.87 (1.72 to 2.02)	< 0.001	1.13 (1.02 to 1.26)	0.023
	secondary	2.72 (2.54 to 2.91)	< 0.001	1.32 (1.18 to 1.47)	< 0.001
	Higher	4.21 (3.71 to 4.78)	< 0.001	1.35 (1.14 to 1.60)	< 0.001
Marital status	Never married	1		1	
	Married	1.01 (0.86 to 1.20)	0.890	1.12 (0.91 to 1.37)	0.28
	separated	1.16 (0.93 to 1.45)	0.190	1.15 (0.88 to 1.50)	0.32
Place of residence	Urban	1		1	
	Rural	0.52 (0.49 to 0.55)	< 0.001	1.05 (0.96 to 1.14)	0.28
Wealth index	Poorest	1			
	Poorer	1.37 (1.27 to 1.49)	< 0.001	1.01 (0.91 to 1.14)	0.91
	Middle	2.32 (2.13 to 2.52)	< 0.001	1.36 (1.22 to 1.52)	< 0.001
	Richer	3.35 (3.06 to 3.70)	< 0.001	1.79 (1.58 to 2.04)	< 0.001
	Richest	4.62 (4.16 to 5.12)	< 0.001	2.32 (1.99 to 2.72)	< 0.001
Region	North central	1		1	
	North east	1.20 (1.10 to 1.31)	< 0.001	1.83 (1.64 to 2.05)	< 0.001
	North west	1.00 (0.92 to 1.09)	< 0.001	1.87 (1.68 to 2.07)	< 0.001
	South east	2.54 (2.26 to 2.85)	< 0.001	1.18 (1.03 to 1.35)	
	South south	1.68 (1.50 to 1.88)	< 0.001	1.64 (1.42 to 1.90)	< 0.001
	South west	1.33 (1.20 to 1.47)	< 0.001	0.61 (0.54 to 0.69)	< 0.001
ANC^a^ visits	No visits	1		1	
	< 4 visits	10.26 (9.31 to 11.30)	< 0.001	9.66 (8.75 to 10.68)	< 0.001
	≥ 4 visits	15.70 (14.51 to 16.98)	< 0.001	14.66 (13.42 to 16.02)	< 0.001
Parity	1 child	1		1	
	2 children	0.97 (0.88 to 1.07)	0.510	1.01 (0.90 to 1.13)	0.89
	≥3 children	0.88 (0.81 to 0.94)	< 0.001	1.08 (0.96 t0 1.21)	0.19

^a^ANC: Antenatal Care

### Inequality in IPTp use

**Fig. 2:**
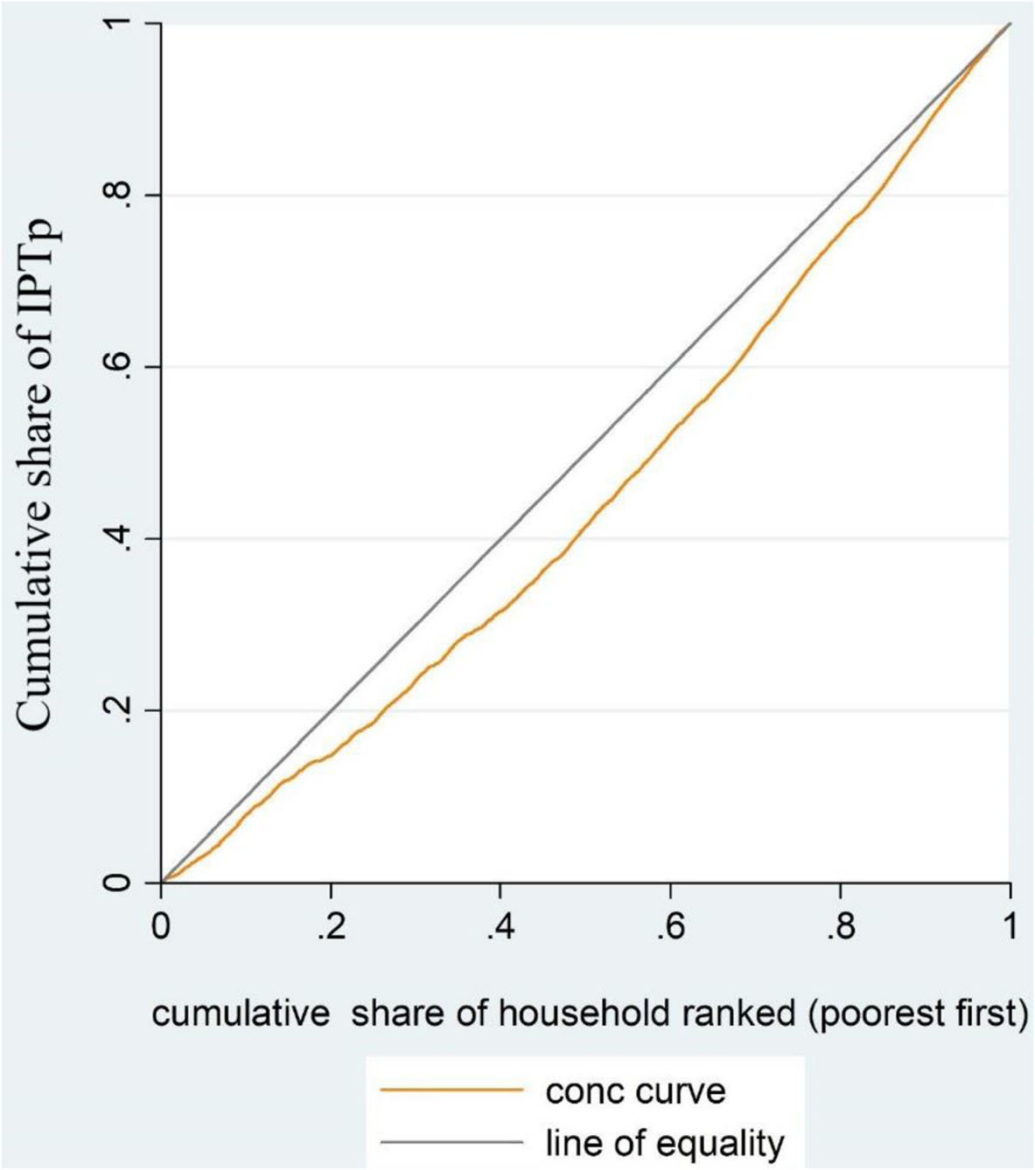
Concentration curve for IPTp (antimalarial drug) among pregnant women. Note: The curve lies below the line of equality refers to pro-rich inequality

IPTp use was pro-rich (Fig. 2) and the concentration index was 0.180 (*p*-value < 0.001) (95% CI:0.176 to 0.183). The Erreyger’s normalized concentration index was 0.280 (*p*-value< 0.001) (95% CI: 0.251 to 0.309) which was significantly different from zero. Wealth index was the main contributor of IPTp use related inequality (47.81) followed by educational status (28.66) (Table 3). Positive and negative signs of the percentage contributed shows the inequality concentrated either in the poorest or richest women (Table 3).

**Table 3:** Decomposing socioeconomic inequality in the use of IPTp among pregnant women Nigeria 2018

Variable	Coefficients	Mean	Elasticity	Concentration index	Absolute contribution	Percentage contribution
Age group						0.69
15–24	1					
25–34	0.018	0.321	0.009	0.083	0.001	0.46
35–44	0.029	0.225	0.010	0.05	0.001	0.30
≥ 45	0.022	0.089	0.003	−0.043	0.000	−0.07
Educational status						28.66
No education	1					
Primary	0.131	0.144	0.030	0.006	0.000	0.10
Secondary	0.177	0.397	0.112	0.282	0.032	17.76
Higher	0.202	0.110	.03	0.540	0.019	10.80
Marital status						−0.18
Never married	1					
Married	0.054	0.696	0.059	−0.008	−0.000	− 0.27
Separated	0.051	0.052	0.004	0.032	0.000	0.09
Place of residence						1.13
Urban	1					
Rural	−0.002	1.541	−0.019	−0.105	0.002	1.13
Wealth Index						47.81
Poorest	1					
Poorer	0.067	0.192	0.020	−0.399	−0.008	−4.50
Middle	0.165	0.196	0.051	0.081	0.004	2.31
Richer	0.221	0.215	0.074	0.407	0.030	17.0
Richest	0.259	0.223	0.092	0.638	0.058	33.0
Region						−9.67
North central	1					
North east	0.148	0.159	0.040	−0.372	−0.015	−8.30
North west	0.119	0.292	0.055	−0.271	0.015	−8.30
South east	0.092	0.119	0.016	0.295	0.004	2.67
South south	0.022	0.116	0.003	0.330	0.001	0.62
South west	−0.049	0.173	−0.014	0.460	0.007	3.64
Parity						**0.03**
1 child	1					
2 children	−0.013	0.165	−0.003	0.120	−0.000	− 0.26
≥ 3 children	−0.008	0.678	−0.009	0.521	0.000	0.29

## Discussion

The results of the current study indicated disproportionate concentration of IPTp-SP intake was pro-rich. Four or more antenatal visit and education status were significantly associated with IPTp utilization in the adjusted odds ratio. This study shows that the pregnant women who took at least one or more doses of IPTp were 63.6%. Among IPTp users, 35.1% took one dose, 38.6% took two doses and 26.2 took three doses and more**.** As recommended by WHO, pregnant women should receive at least 3 doses of Sulphadoxine pyrimethamine, which was revised to a monthly administration during pregnancy [19], necessitating the need to increase its access. Recent studies in Ghana and Malawi reported three or more doses to be 64.5 and 70.2%, respectively [20, 21]. Although the coverage of at least one dose of IPTp use has increased from 27% in 2013 to 64% in 2018, the coverage of three or more doses is much lower than other studies [14]. Even for the uptake of at least one dose of IPTp, the Nigerian Demographic & Health Survey reports variation among pregnant women in urban and rural areas (72.6 & 58.0% respectively) [14].

The value of the concentration index of IPTp intake was 0.180, indicating an increased use of IPTp among the rich. Further, decomposition of IPTp revealed that the variables wealth index and the level of education as the main contributors. On the other hand, age, marital status, place of residence, region and parity had insignificant influence to the observed socioeconomic inequality. Another study reported from earlier work in Nigeria, using only the concentration index to assess inequality, also showed the use of IPTp utilization as pro-rich [13]. This finding was comparable with the study reported from Kenya [22] which shows that poor individuals were less likely to use any kind of antimalarial drugs for pregnant women. In other studies, in some developing countries, IPTp use during pregnancy was concentrated among women in the richest households [8, 23, 24].

In contrast, the study reported by Mathanga et al. [12] revealed no inequality between pregnant women on IPTp utilization. The difference between our finding and previous study results is probably because antenatal attendance is very high in Malawi across the socioeconomic quintiles, providing a great opportunity to reach all pregnant women with IPTp, unlike in Nigeria where there is stock out of the drug as reported in some studies [25].

The adjusted odds ratio showed that covariates such as higher educational status and adequate antenatal visits significantly contributed to the IPTp utilization during pregnancy in Nigeria. Women with secondary and higher education had higher odds of taking IPTp-SP compared to those with no education. This reveals that educated women are aware of the effect of malaria in pregnancy, consistent with other studies that showed educated women are more likely to take IPTp-SP [26, 27]. The number of ANC visits was significantly associated with at least one dose of IPTp-SP. This is not surprising because pregnant women are recommended to be given the drug during the ANC visits [4]. Although findings from a systematic review shows inconsistent association between the ANC attendance and the IPTp uptake [28], the possible reason of the variation is that some women attend ANC but were not given SP due to stock out [25, 29]. Due to the high correlation observed between ANC visits and IPTp in this study, the ANC variable was excluded during the decomposition analysis.

The odds of taking a dose of SP among women with high parity (three or more children) was lower in the bivariate analysis. However, the association was no longer significant after adjusting for education status, age, marital status, region, antenatal visits and wealth index. Ideally women with more children should have known the importance of IPT due to previous pregnancies. This is in consonance with a study by Bouyou-Akotet et al. which shows that having more than four children lowers the intake [30]. This result contradicts a study in Uganda which reported that women with more children used IPTp due to the possible exposure to the message of its significance [31]. Age, marital status and place of residence were not significant in the multiple regression but age and place of residence were significant in the univariate model.

The present study has some limitations. The cross sectional nature of the study design could not show the causal relationship between the available inequality on IPTp utilization and the factors that contributed to the inequality. In addition, all potential determinant factors of inequality were not included in the analysis. This might limit the comprehensiveness of our findings on the observed inequality.

## Conclusion

The current study revealed considerable inequality between pregnant women in IPTp utilization in Nigeria. IPTp use is concentrated among women from the richest households which are more likely to take one or more dose compared to their counterparts. Free IPTp service expansion through targeting pregnant women from low socioeconomic status and rural area are important to reduce the available inequality. Policy makers might wish to implement other effective delivery method such as the community based delivery approach for IPTp. Further research is needed to explore the barriers of IPTp intake in Nigeria.

## Supplementary Information

**Additional file 1: Supplementary Table S1.** Explanatory variables included in the decomposition analysis.

## Data Availability

The data for this work can be accessed on the DHS website. Available at: https://dhsprogram.com/data/dataset/Nigeria_Standard-DHS_2018.cfm?flag=1.

## Abbreviations

ANC: Antenatal care
IPTp: Intermittent preventive treatment in pregnancy
NDHS: Nigerian demographic health survey
SP: Sulphadoxine Pyrimethamine
WHO: World health organization
