# Supplementary material for: Socioeconomic inequality in intermittent preventive treatment using Sulphadoxine pyrimethamine among pregnant women in Nigeria
Source: BMC Public Health. 2020 Dec 4;20:1860. doi: 10.1186/s12889-020-09967-w (PMC7716500; doi:10.1186/s12889-020-09967-w)
Supplement: Supplementary file 1 — Additional file 1: Supplementary Table S1. Explanatory variables included in the decomposition analysis. [file 12889_2020_9967_MOESM1_ESM.docx]

# Supplementary Materials

Supplementary Table 1: Explanatory variables included in the decomposition analysis

| Variable | Category |
| --- | --- |
| Age group | Women current age category  15-24yrs=1,25-34yrs=2,35-44yrs=3, ≥45=4 |
| Place of residence | Urban=1 Rural=2, |
| Educational status | Highest educational level attained  No education=0, Primary=1, Secondary=2, Higher=3 |
| Marital Status | Never married =0, Married= 1, Separated=2 |
| Parity | Number of live births a woman.  1 child=1, 2children=2, ≥3children=3 |
| Wealth index | DHS program provides a composite index based on the principal component analysis of household amenities as wealth index. We categorized this index into five groups: 1st quintile (Poorest); 2nd quintile; 3rd quintile; 4th quintile and 5th quintile (Richest) as provided by the DHS data set . |
| Region | North central=1, North east=2, North west=3, South east=4, South South=5, South west=6 |
